# CRPV Genomes with Synonymous Codon Optimizations in the CRPV E7 Gene Show Phenotypic Differences in Growth and Altered Immunity upon E7 Vaccination

**DOI:** 10.1371/journal.pone.0002947

**Published:** 2008-08-13

**Authors:** Nancy M. Cladel, Jiafen Hu, Karla K. Balogh, Neil D. Christensen

**Affiliations:** 1 Jake Gittlen Cancer Research Foundation, Pennsylvania State University College of Medicine, Hershey, Pennsylvania, United States of America; 2 Department of Pathology, Pennsylvania State University College of Medicine, Hershey, Pennsylvania, United States of America; 3 Department of Microbiology and Immunology, Pennsylvania State University College of Medicine, Hershey, Pennsylvania, United States of America; The Rockefeller University, United States of America

## Abstract

Papillomaviruses use rare codons relative to their hosts. Recent studies have demonstrated that synonymous codon changes in viral genes can lead to increased protein production when the codons are matched to those of cells in which the protein is being expressed. We theorized that the immunogenicity of the virus would be enhanced by matching codons of selected viral genes to those of the host. We report here that synonymous codon changes in the E7 oncogene are tolerated in the context of the cottontail rabbit papillomavirus (CRPV) genome. Papilloma growth rates differ depending upon the changes made indicating that synonymous codons are not necessarily neutral. Immunization with wild type E7 DNA yielded significant protection from subsequent challenge by both wild type and codon-modified genomes. The reduction in growth was most dramatic with the genome containing the greatest number of synonymous codon changes.

## Introduction

Papillomaviruses are double-stranded DNA tumor viruses of about 8 kb. There are more than 100 human papillomavirus types, some specific for mucosal tissues and others for cutaneous sites. A subset of the viruses is associated with cancers, in particular cancer of the cervix.

Papillomaviruses use rare codons relative to their hosts [1], [2]. It has been argued that this is an evolutionary adaptation, which allows the virus to escape immune surveillance [3]. Nonetheless, the reasons for codon bias are poorly understood. There is a growing recognition that synonymous codon usage is not always neutral [4]–[6]. Many reasons have been posited for codon choice. Among them are: **1.** Control of translation rate and gene expression [7], [8]. **2.** Control of protein folding [9]–[11]. **3.** Nucleosome positioning [12]. **4.** Tissue specificity requirements [13]–[15]. **5.** RNA stability, secondary structure and degradation parameters [16]–[20] **6**. Selection for translational efficiency [21] and **7.** Provision for splicing [22].

There are very few models to study the impact of synonymous codon changes. Carlini [23] changed common leucine codons to uncommon counterparts in the alcohol dehydrogenase gene of Drosophila and found decreasing tolerance to alcohol with increasing numbers of uncommon codons. Komar et. al. [10] replaced 16 rare codons in the chlorampenicol acetyltransferase (CAT) gene of *E. coli* with common codons and found accelerated protein synthesis but reduced specific activity. The authors interpreted their data to suggest that the accelerated rate of protein production resulted in partial misfolding of the protein and hence, reduced activity. Poliovirus capsid gene deoptimization, reported in two recent studies, led to attenuation of the virus [24], [25]. While the two groups did not agree on the mechanisms for the attenuation, their results are in agreement and show the power of synonymous codon change to produce phenotypic changes in the virus.

A major focus of this laboratory is the immunology of papillomaviruses. Our previous studies as well as those of others have shown that vaccination of the domestic rabbit with the four early proteins of the cottontail rabbit papillomavirus(CRPV) (E1, E2, E6 and E7) can provide complete protection from subsequent viral challenge [26], [27]. We have also shown that E7 does not appear to participate in this protection although vaccination with E7 does delay progression to cancer [28]. E7 is one of the oncogenes of the papillomavirus. Because of its continuous presence in progressive tumors, E7 has been tested as an immunotherapeutic target in animal models and clinical trials [29]–[32]. We have recently begun studies with this model to examine host-virus interactions following synonymous codon alterations in several viral genes. In our initial study reported here, we hypothesized that codon “optimization” of the E7 gene in the context of the CRPV genome would result in increased protein production and thus enhanced immunogenicity. To investigate this question, we constructed E7 genes with 8, 14, 18 and 22 synonymous codon changes (E7/8, E7/14 etc.). *In vitro* studies with these genes confirmed that more E7 protein was produced by E7/18 and E7/22 than by E7/wild type(wt), E7/8 or E7/14. The increased protein production could have resulted from the increased mRNA (E7/22) and/or the more stable mRNA (E7/18 and E7/22) that was demonstrated by QRT-PCR analysis. We placed these modified E7 genes into the CRPV genome in place of the wild type E7 gene. These codon-modified genomes were tested for functional growth and for immunogenicity following E7 vaccination. We found that all genomes were functional and all responded by growth reduction upon immunization with wild type E7 DNA vaccine. CRPV containing 22 changes in E7 showed the largest growth retardation in vaccinated animals. This work demonstrates the concept that codon bias can play a role in the immune response to an infectious organism and supports the hypothesis that papillomaviruses have evolved their codon usage to reduce exposure to immune detection. Our *in vivo* model will be a useful tool to study further questions surrounding papillomavirus codon bias in the papillomavirus life cycle.

## Results

### Protein production, as determined by immunoprecipitation, increased with increasing numbers of optimized codons in E7

**Figure 1:**
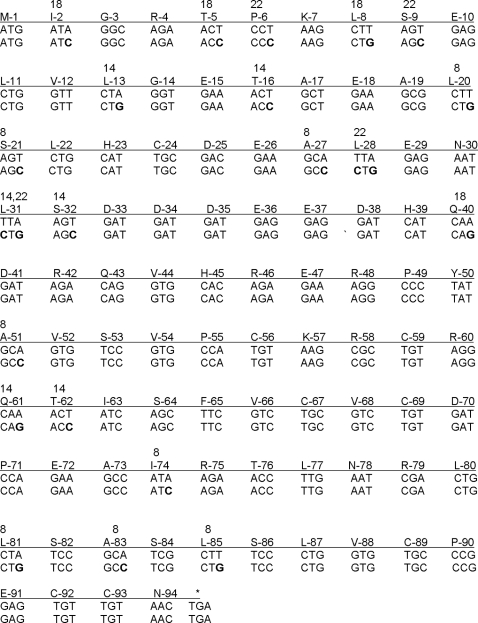
Location of codon changes in H. CRPV E7. Each of the codon changes introduced into H. CRPV E7 is noted. The changes were cumulative. For example E7/14 contains all the changes in E7/8 plus 6 unique to itself; E7/18 contains the 14 in E7/14 plus 4 unique to itself; E7/22 contains all of the changes noted. Upper lines represent wild type E7 sequence and lower lines, the codon-modified sequences with changes noted in bold. Single letter notations are used for the amino acids. The numbers above selected amino acids identify the genome containing the codon modification of that amino acid.

**Figure 2:**
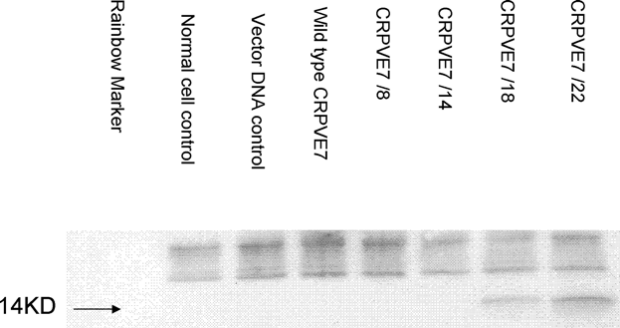
Immunoprecipitation of E7 proteins. Protein from transiently transfected cells was immunoprecipitated, run out on a PAGE gel and detected using a monoclonal antibody to E7, MAb E7-1. Increasing amounts of protein were detected with increasing numbers of codon changes.

Codon changes within the CRPV E7 gene were made to match common codon usage of mammalian genes. A series of 4 E7 genes was constructed to give 8, 14, 18 and 22 sequential codon changes (Fig 1). The latter 3 constructs were built upon the changes already incorporated into the previous construct. This strategy was designed to generate a panel of intermediate reagents that could be used to test the effects of codon changes and also to detect possible lethal genetic effects that could potentially occur if all codons were changed in a single and final E7 construct. The E7 protein produced in transiently transfected cells was immunoprecipitated using a monoclonal antibody to CRPV E7 (E7-1) generated in house. Protein was detected in the positive control and in cells transfected with E7/18 and E7/22 but not in cells transfected with wild type E7 or E7/8 and E7/14 (Fig 2). These *in vitro* results are consistent with the hypothesis that increases in “optimal” codons may favor increased protein production and also consistent with the work of Cid-Arregui, et. al. with HPV 16 E7 [33]. In addition, Samorski et. al. [34] report similar findings with HPV 16 E6.

### Increased E7 protein production with increasing numbers of codon “optimizations” was confirmed by FSCAN analysis

**Figure 3:**
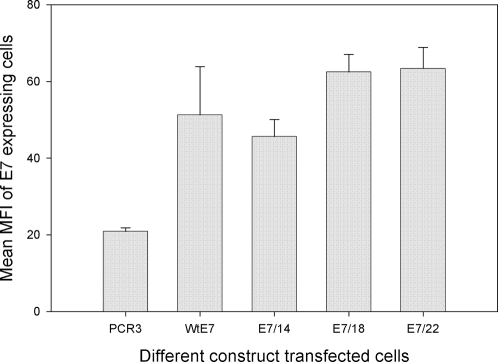
FSCAN analysis of cells transfected with E7 expression constructs. Expression of E7 protein was detected in immortalized rabbit cells transfected with expression constructs of the E7 genes and subjected to FSCAN analysis. All analyses were done in duplicate. E7/18 and E7/22 showed the highest expression levels.( p<0.05, unpaired student t test), when compared to the control.

**Figure 4:**
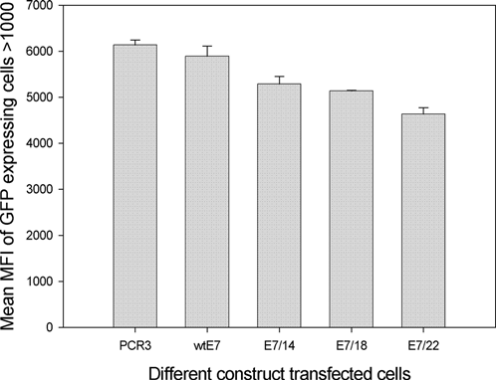
Analysis of expression of EGFP, used as a control for transfection efficiency. Expression of EGFP was detected in all cells cotransfected with E7 genes and an EGFP expression construct. All transfections were done in duplicate. The amount of protein expressed declined with increasing numbers of optimized codons in the E7 genes suggesting down regulation due to expression of E7 (p<0.05, unpaired student t test).

E7 expression in cell lines transfected with wild type E7, E7/14, E7/18, and E7/22 was also tested by FSCAN flow cytometry. Consistent with the results from the immunoprecipitation study, statistically significant increase in protein expression with increasing numbers of codon changes was found (Fig 3, P<0.05, unpaired student t test). The increased protein production appeared to suppress the expression of EGFP significantly when EGFP was co-transfected at the same time as a control for transfection efficiency (Fig 4, P<0.05, unpaired student t test).

### E7 Messenger RNA levels were elevated in transiently but not in stably transfected cells

**Figure 5:**
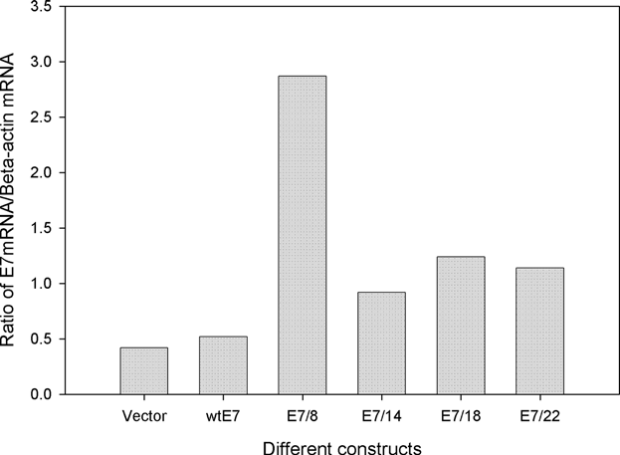
Northern blot analysis of cells transiently transfected with E7 expression constructs. RNA from transiently transfected cells was blotted to Hybond N^+^ membrane and probed for E7 message using a mixture of all E7 genes for the probe. Message was standardized to beta actin control. Elevated E7 RNA signals were obtained for all codon-modified genes; E7/8 gave the highest signal.

To investigate whether E7 protein levels correlated with RNA levels, Northern blot analysis was performed. RNA extracted from cell lines transiently transfected with expression constructs of the various optimized E7 genes showed a somewhat elevated expression over wild type in the two analyses conducted (Fig 5 is representative). E7/8 showed the greatest elevation in both studies undertaken. Northern analysis on RNA from stably transfected cell lines, however, showed very little message. Whereas control β-actin expression level was strong at six hours, a 96-hour exposure was necessary to detect the very weak E7 signals (data not shown). We conclude that there is selection pressure in cells containing E7 DNA such that stable lines express a limited amount of E7 protein. However, in transiently transfected cells and prior to integration, the cells do produce E7 message which appears to be elevated in codon-optimized genes, especially E7/8. Similar findings were reported by Cid-Arregui, et al. [33].

### Quantitative RT- PCR analysis on mRNA isolated from transiently transfected cells treated with Actinomycin D at different time points showed elevated message for E7/22 and more stable message for E7/18 and E7/22

**Figure 6:**
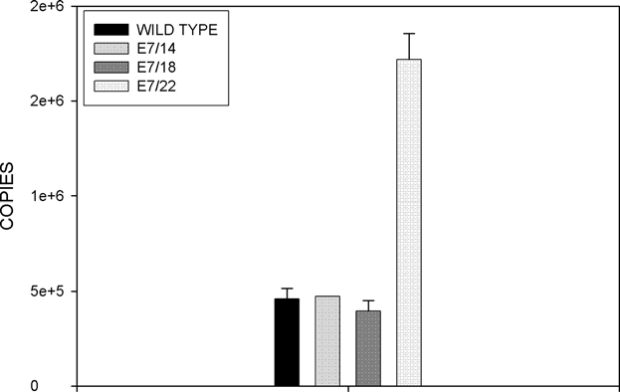
Quantitative RT PCR analysis on total RNA from cells transiently transfected with wild type E7 or codon-modified E7 expression constructs demonstrated that E7/22 message was present at about four times the numbers of copies of the other genes. RNA was isolated from transiently transfected cells and subjected to QRT- PCR. Standard curves were run with each reaction and quantitation of message was done using these curves. E7/22 message was present at levels four fold higher than the other genes.

**Figure 7:**
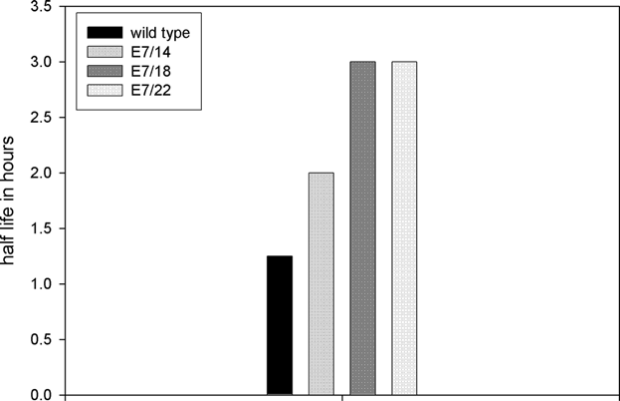
An Actinomycin D time course demonstrated that message stability increased with increasing numbers of codon changes. RNA was isolated from transiently transfected cells at different time points from 0 to six hours post treatment. RNA stability increased with increasing numbers of codon changes.

Quantitative RT- PCR( QRT-PCR) is a powerful tool for the quantitative analysis of transcripts. We chose to follow up the Northern analysis above with the far more sensitive quantitative RT-PCR analysis and to combine this with an Actinomycin D assay in order to evaluate stability of the messages over time. In this study we looked at wild type E7 and E7/14, E7/18, and E7/22. E7/8 was not included due to assay constraints. The TBP (TATA binding protein) was used as internal standard to make use of primers and probes available in the laboratory. All codon-modified genes utilized the same set of E7 primers and probe; E7/wild type amplification required a slightly modified set. In this analysis, which was conducted in duplicate, we found that TBP was not stable over time; thus each analysis included a standard curve to allow for quantitation. We found that E7/wild type, E7/14 and E7/18 messages were all present at about the same level whereas E7/22 message was four fold higher (Fig 6). Furthermore, we determined that message stability increased with increasing numbers of codon changes (Fig 7). The relative mRNA values were not entirely consistent with those obtained by Northern analysis above. The differences may be attributable to different conditions in the respective assays and/or to the quantitation methods. The Northern analysis was quantitated by densitometry, which is inherently less reliable than the QRT-PCR analysis, in part due to potential saturation of signals. Of note is the fact that E7/8, whose message was most highly elevated in the Northern analysis, was not able to be included in the QRT-PCR analysis. This will need to be addressed in future experiments.

### RNA extracted from papillomas generated by codon-optimized genomes did not show elevated message

Northern blot analysis was also conducted on RNA extracted from selected papillomas generated by both wild type and codon-optimized genomes. No apparent difference in E7 RNA level was found among these samples suggesting that codon optimization may not significantly influence E7 message production in the intact host (data not shown). Message and protein production are not always correlated and so we cannot conclude that the failure to detect differences in RNA quantity *in vivo* implies that there are no differences in protein production.

### CRPV genomes, containing E7 with codon optimizations, were functional and phenotypes were different

**Figure 8:**
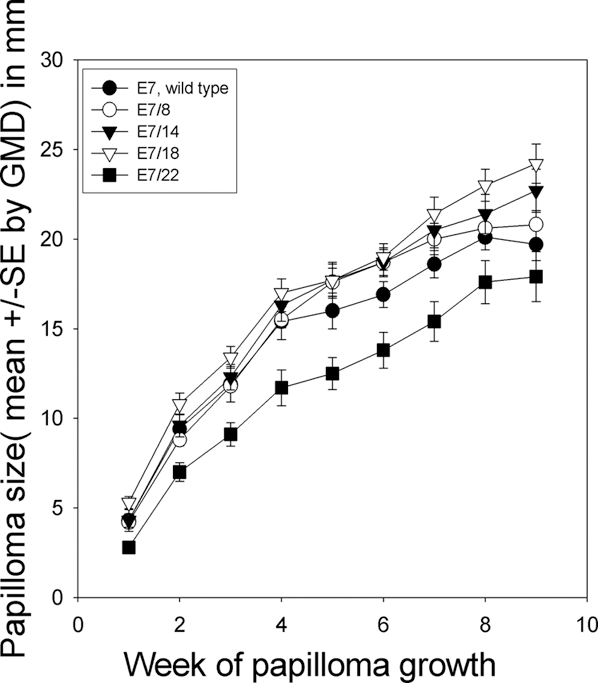
Sizes of papillomas differed among the different genotypes. Growth curves of papillomas following infection with wild type and codon-optimized genomes. E7/8 and E7/14 papillomas grew at about wild type rate; E7/18 papillomas grew statistically faster than E7/22 papillomas (P<0. 001, unpaired student's t test); E7/18 papillomas grew statistically faster at later time points than wild type papillomas (P<0.03 unpaired student's t test).

All genomes containing E7 optimizations were tested in New Zealand White (NZW) outbred rabbits using our DNA infectivity assay. All constructs were able to induce papillomas in these animals at high efficiency. Papilloma growth occurred at wild type rate for genomes containing E7/8 and E7/14, at an accelerated rate for the genome containing E7/18, and at a reduced rate for the genome containing E7/22 (Fig 8). There was a highly significant difference between the growth rates of E7/18 and E7/22 genomes (p<0.001, unpaired student t test) and a moderately significant difference between E7/18 and wild type at later time points (p<0.03, unpaired student t test; Fig 8). There was a significant difference in growth rates at most time points between E7/wild type and E7/22 ( p<0.02). These results demonstrate that codon changes in the viral DNA are not necessarily neutral.

### Codon-optimized changes were maintained in papilloma DNA extracted from tumors

Genomic DNA was extracted from papillomas initiated with different codon-optimized constructs and PCR was performed to amplify the E7 region. The amplimers were sequenced. In all cases investigated, the changes that had been introduced were maintained in the replicated DNA (data not shown).

### E7 DNA immunization resulted in a statistically significant reduction in the growth of papillomas induced by codon-optimized constructs

**Figure 9:**
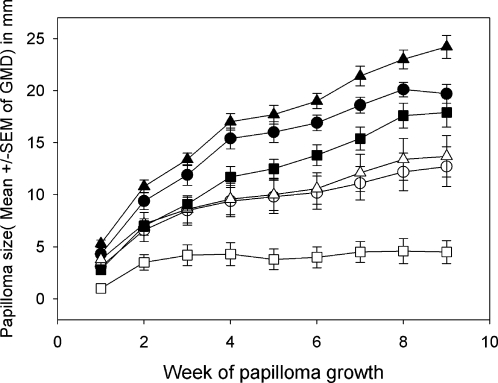
Results of vaccination study. Animals were vaccinated with either wild type E7 (◯,▵,□) or empty vector (•,▴,▪) and were then challenged with each of the CRPV genomes including wild type. Vaccination resulted in a reduction in size of all papillomas. Volumes of wild type, E7/8, E7/14 and E7/18 papillomas were reduced 30–50%, and volumes of E7/22 papillomas were reduced 75% (see Table 1). Figure shows results for wild type (◯,•), E7/18 (▵,▴) and E7/22 (□,▪) papillomas.

An immunization experiment was undertaken to explore the response of codon-modified genomes to E7 DNA immunization. Following a series of four gene gun immunizations with either wild type E7 plasmid, or vector only, six animals per group were challenged at two sites each with wild type CRPV DNA and with each of the codon-optimized DNAs. Papilloma growth was monitored for a period of two months. Complete protection was seen for the E7 immunogen in one out of six rabbits. Volume of all E7/8, E7/14 and E7/18 papillomas in the E7 immunogen group was reduced by 30–50% when compared to those in the vector immunization group, whereas volume of E7/22 papillomas was reduced by 75%. (Fig 9, Table 1). These data support the possibility that E7/22 is more immunogenic because the codon optimizations present allow for the production of more protein.

**Table 1:** E7 immunization resulted in reduced papilloma size.

Genome	% reduction in papilloma size by E7 immunization	
	week 8	week 11
Wild type	45%	36%
E7/8	40%	30%
E7/14	38%	31%
E7/18	53%	43%
E7/22	74%	75%

Immunization of animals with wild type E7, followed by infection with wild type and four codon-optimized genomes, resulted in reduction in size of papillomas. Two time points (8 and 11 weeks) after infection are presented. The effect was most notable with E7/22 for which a 75% reduction in size was measured.

## Discussion

Papillomaviruses show significant bias in their codon usage with a preference for AT_3_ vs. CG_3_ [40]. In an effort to investigate the significance of this bias, and in acknowledgement of recent work, which has shown increased protein production *in vitro* using codon-optimized genes [2], [41], [42], we used our rabbit/CRPV DNA infectivity model to allow us to begin to study synonymous codon usage via alterations in the CRPV genome. We postulated that modifying viral codons to be more mammalian-like would result in increased protein production and that this change could be assessed immunologically.

The CRPV model is the only animal model in which the entire life cycle of a papillomavirus from initiation of infection to malignant progression can be studied [27], 28, 39. The model has the advantage that viral DNAs are infectious and thus mutants can be made and evaluated without the need to produce infectious virions. Synonymous codon mutants of the oncoprotein E7 were developed to investigate the immunological response of genomes containing these modifications. We chose the E7 protein for our target as our earlier work had shown that the wild type protein was poorly immunogenic [28]. Codon modifications of HPV16 E7 have been shown to enhance cytotoxic T cell Lymphocyte ( CTL) induction and antitumor activity[32]. We hypothesized that increasing numbers of “optimizations” of CRPV E7 within the CRPV genome would lead to increased protein production and that this would be detected as an improved response to immunization. This approach is unique in that the infectious agent was codon-modified rather than the immunogen, as is typically done.

We have shown in this study, using an *in vitro* assay, that increasing numbers of optimizations did, in fact, lead to increased protein production. However, *in situ* quantitation of E7 content in papillomas is more difficult and we do not yet have definitive proof that this also occurred *in vivo.* We have shown, for example, that significant protection was achieved when wild type E7 immunization was followed by challenge with both wild type and codon-modified genomes on the same animal. In our previous work, no protection was found with wild type E7 immunization when animals were subsequently challenged with wild type CRPV [28]. We interpret these results to suggest that one or more of the codon-modified genomes produced enough E7 protein to be detected by the immune system and that this stimulated a systemic response that was manifested as a reduction in size of papillomas initiated by all genomes including wild type.

We created four mutant viral strains containing 8, 14, 18, or 22 synonymous changes in E7. Changes were made at random to codons for a number of amino acids including isoleucine (2), threonine (3), proline (1), leucine (7), serine (2), glutamine (2), and alanine (3). CG_3_ for wild type H. CRPV E7 is 44%, whereas CG_3_ for our most highly optimized genome, E7/22, is 65%. Thus, the changes yielded E7 genes whose codons more closely approximated those of mammals as reported by Zhou et. al. [2]. We compared the ubiquitously expressed MHC class I rabbit gene sequence (Genbank K024441) with a BLAST search of human genes and found greater than 90% homology between human MHC class I precursors and the rabbit gene. Codon usage is nearly identical. This lends validity to our appropriation of human codon usage in the absence of sufficient information on the rabbit genome. We should note that we made codon changes based on the most common usage on average in the human genome. We acknowledge that we do not know if these codons are truly “optimal”, that is selected for maximal translation in mammals. The understanding of codon usage, especially in mammalian systems, is still incomplete. We can simply say at this point that we replaced codons of CRPV E7 , codons known to be uncommon in humans , with ones most commonly used in humans, a technique that has been shown to greatly increase protein production *in vitro.*

We were concerned that the codon changes might interrupt cryptic regulatory regions in the gene and so we built the genes sequentially in order to be able to detect this possibility. We found that the first two changes, E7/8 and E7/14, resulted in no obvious phenotypic differences. The third change, E7/18, yielded a somewhat enhanced growth rate and the fourth, E7/22, a significantly reduced growth rate. The differences between the E7/18 and E7/22 growth rates were highly significant (p<0 .001, Student's unpaired t test). We note that while our codon changes were made at random, the end result was not random in that the final changes (E7/18 and E7/22) were clustered in the 5′ region of the gene. We cannot rule out the possibility that the phenotypic differences seen with these two genomes were the result of a special sensitivity to change in this early region of the gene. This possibility will be investigated in future experiments.

If protein expression increases with increasing numbers of codon optimizations in E7, we might expect three possibilities. As mentioned above, we might expect enhanced immune surveillance and concomitant reduced growth. Alternatively, we might expect increased growth due to enhanced activity of the oncogene. A third possibility would be reduced growth rate due to excess E7 oncoprotein leading to increased apoptosis [43]. This apoptotic effect could be due to either direct toxicity of the E7 protein or to stimulation of cellular immunity which, in turn, would stimulate apoptosis. All three mechanisms may counter-balance each other *in situ* with growth being kept in check by immune control and/or apoptosis, or enhanced by increased oncogenic activities. The data in this paper would support this dynamic tension with E7/18 genomes demonstrating more oncogenic effects and E7/22 genomes inducing more immunological responses. These scenarios are supported both by the growth data in the absence of immunization and by the immunization study in which greatest reduction of papilloma growth was observed in the E7/22 genome.

To our knowledge, this is the first *in vivo* study using codon-altered papillomavirus genomes. We have shown phenotypic differences in growth rates resulting from the changes as well as differential responses to immunization. The work supports the increasing body of evidence that synonymous codons are not always silent and paves the way for further studies on codon selection in E7 and other papillomavirus genes. We anticipate that our model will be useful in helping to dissect the significance of alternative codon usages.

## Materials and Methods

### Construction of codon-modified genomes

The Hershey CRPV progressive strain (H. CRPV) was used as the backbone for all constructs. It was cloned into PUC 19 at Sal1. The EcoRI site in the vector was removed and a Cla1 site was introduced at 1383 just downstream of the E1 start. This site, in conjunction with the naturally occurring EcoR1 site at 1063, was used to replace wild type E7 with codon-modified E7 genes.

Codon-modified E7 genes were created by site-directed mutagenesis of wild type E7 cloned into PUC 19 at EcoR1 and BamH1. The E7 gene with BamH1 and EcoR1 ends was obtained by PCR of the modified CRPV genome containing the Cla1 site and this allowed for placement of modified E7 genes into the wild type backbone. Codon changes were based on the work of Zhou et. al. [2] and were done at random. Each construct contained all mutations from the previous one; thus E7/14 contained all the codon changes in E7/8 plus six more; E7/18 contained those 14 codons plus 4 more, etc. Mutagenesis was carried out using Pfu turbo (Stratagene, LaJolla, CA) and a modification of the protocol for the Quick Change site directed mutagenesis method in which the first round of amplification was done with a single primer [35]. All mutations were verified by DNA sequencing at the Core Facility of Pennsylvania State University College of Medicine. Mutations are shown in Table 2 and Fig 1.

**Table 2:** Codon changes were done at random and include those for numerous amino acids spanning the entire E7 gene.

2	isoleucine	ata>atc	E7/18
5	threonine	act>acc	E7/18
6	proline	cct>ccc	E7/22
8	leucine	ctt>ctg	E7/18
9	serine	agt>agc	E7/22
13	leucine	cta.>ctg	E7/14
16	threonine	act>acc	E7/14
20	leucine	ctt>ctg	E7/8
21	serine	agt>agc	E7/8
27	alanine	gca>gcc	E7/8
28	leucine	tta>ctg	E7/22
31	leucine	tta>cta	E7/14
31	leucine	cta>ctg	E7/22
32	serine	agt>agc	E7/14
40	glutamine	caa>cag	E7/18
51	alanine	gca>gcc	E7/8
61	glutamine	caa>cag	E7/14
62	threonine	act>acc	E7/14
74	isoleucine	ata>atc	E7/8
81	leucine	cta>ctg	E7/8
83	alanine	gca>gcc	E7/8
85	leucine	ctt>ctg	E7/8

The location (amino acid number within the E7 gene) and identity of all codon changes is shown as well as the amino acids whose codons were changed. Genomes were built sequentially. Thus, E7/14 contains all the changes in E7/8 plus 6 unique to itself. E7/18 contains all E7/14 changes plus 4 unique to itself. E7/22 contains all of the previous changes plus 4 unique to itself.

### Construction of immunogen and expression constructs

Wild type CRPV E7 cloned into the VIJns expression vector as previously reported was used as immunogen for DNA vaccination [26]. VIJns empty vector was used as control. Wild type CRPVE7, E7/8, E7/14, E7/18 and E7/22 were also cloned into an expression vector PCR3 (Invitrogen) for testing of *in vitro* expression. All constructs were confirmed by sequencing in the Core facility of Pennsylvania State University College of Medicine.

### E7 detection by flow cytometry analysis

One µg of PCR3 containing wtE7, E7/8, E7/14, E7/18 or E7/22 was co-transfected with 0.5 µg PCR3 containing EGFP into an immortalized rabbit cell line (RI) cultured in a 6-well plate (samples in duplicates). The cells were harvested 48 hrs after transient transfection and examined for GFP and nuclear E7 expression. For GFP expression, the cells were fixed with 1×PBS containing 2% Paraformaldehyde (PFA); For E7 expression, the cells were fixed with cytofix/cytoperm (BD) solution and incubated with an in-house generated mouse anti CRPVE7 monoclonal antibody (SE7.1, 1∶500) and subsequently with a PE-conjugated anti-mouse IgG (1∶50). The cells were then fixed with 2% PFA PBS for FSCAN test as previously described [36].

### E7 detection by immunoprecipitation

Cells (1×10^6^) transiently transfected with the different E7 constructs were also harvested in 500 µl lysis buffer (RIPA: 50 mM Tris.HCl PH 7.5, 150 mM NaCl, 1% NP-40, 0.5% Sodium Deoxycholride, 0.1% SDS) on ice for 30 minutes. The cells were spun down (14,000 rpm, 10 minutes). 100 µl of the supernatant was incubated with 5 µl of normal rabbit serum and 20 µl of sepharose A and rotated in the cold room (4°C) for 2 hours. The mixture was spun down again and the supernatant was collected and incubated with 20 µl of sepharose A and mouse anti-CRPVE7 MAb (1∶1000) overnight in a cold room rocker. An additional 30 µl of sepharose A was added the next day and incubated for 2 more hours before the cells were spun down. The pellets were collected and boiled with 40microliters protein loading buffer for five minutes. The samples were analyzed via western blot assay as previously described [37].

### Northern blot analysis

RNAs were isolated from either transfected cells or papilloma tissue using the Trizol reagent (Invitrogen, Carlsbad, CA). 5 µg RNA from each sample (duplicates for each sample) was run out on a 1% formaldehyde gel using reagents from the Ambion Northern Max kit (Ambion, Austin, Texas). RNAs were blotted to Hybond N^+^ (Amersham, Piscataway, N.J.) using downward transfer and were fixed to the membrane using the Stratalinker 1800(Stratagene, La Jolla, CA). ^32^P ATP probe was prepared from a mixture of wild type and codon-modified DNAs using standard Klenow labeling. Hybridization was carried out according to the Ambion protocol. Membrane was exposed to Kodak X-OMAT film (Rochester, NY). Densitometric analysis to determine relative signals was done in the Core Facility at Penn State University College of Medicine. Membranes were stripped following probing with the labeled E7 DNA mixture and reprobed with beta actin as a loading control.

### Quantitative RT-PCR analysis of total RNA isolated from cells transiently transfected with expression constructs of wild type E7, E7/14, E7/18 and E7/22 and isolated at different time points following treatment with Actinomycin D

To evaluate mRNA copy number and mRNA stability and to look at these parameters over time, we did QRT- PCR analysis on RNAs extracted from cells that had been transiently transfected with E7 expression constructs and isolated at time points 0 hours, one hour, 1.5 hours, 2 hours, 4 hours and 6 hours following treatment with Actinomycin D. RNAs were isolated using the TRIzol (Invitrogen) reagent and were treated with Turbo-DNA-free (Ambion) to remove any contaminating DNA; they were then quantitated spectrophotometrically. Reverse transcription and quantitative PCR were performed on 100 ng total RNA per reaction in the same closed strip tubes using Stratagene's Brilliant II QRT-PCR one step kit (# 600809). Primers and probe were designed using Primer Express software^©^. TATA-binding protein (TBP) amplicons were generated using primers 5′ CACGGCACTGATTTTCAGTTCT 3′ (nt 627–648) and 5′TTCTTGCTGCCAGTCTGGACT 3′ (nt706–686) at final concentrations of 200 nM `. Probe for TBP was fluorogenic TaqMan probe 5′HEX TGTGCACAGGAGCCAAGAGTGAAGA BHQ-1 3′ and was used at a final concentration of 100 nM. CRPV E7/14, 18 and 22 primers were 5′AAGCGCTGTAGGCAGACCA 3′ (N 16–35) and 5′TCGATTCAAGGTTCTGATGGC 3′ (84–105) and were used at a final concentration of 300 nM. Probe was fluorogenic TaqMan probe 5′FAM CAGCTTCGTCTGCGTCTGTGATCCA-BHQ-1 3′ (36–61) and was at a final concentration of 100 nM. Wild type CRPV E7 primers were 5′ CCATGTAAGCGCTGTAGGCAAA 3′ (10–32) and 5′GCAGTCGATTCAAGGTTCTTATGGC 3′ (85–109) and were used at a final concentration of 300 nM. Probe was 5′FAM TCAGCTTCGTCTGCGTCTGTGATCC BHQ-1 3′ (35–60) and was used at a final concentration of 100 nM. Primers and probes for the E7 genes were synthesized by Integrated DNA Technologies. A standard curve was run on each plate using the appropriate E7 plasmid DNA as template at 1∶2 dilutions. All QRT- PCR reactions were performed using the Mx-4000 (Stratagene). Cycling conditions were 50°C for 30 min (reverse transcription) followed by one cycle at 95°C to activate the polymerase, and then by 42 cycles of 94°C for 15 sec and 60°C for one min. Absolute quantities were determined using the standard curve. Each transfection was carried out in duplicate and RNAs from each time point for each E7 gene were run in duplicate. Each gene required a separate run. No template (No NT) controls were included on all plates and No reverse transcriptase (No RT) reactions were run for all samples. Standard curve reactions did not receive reverse transcriptase.

### DNA vaccination of rabbits

New Zealand White (NZW) outbred rabbits were purchased from Covance (Denver, PA) and maintained in the animal facility of the Pennsylvania State University College of Medicine as described above. The studies were approved by the Institutional Animal Care and Use Committee of the Pennsylvania State University. Inner ear skin sites were shaved and swabbed with 70% ethanol, and then DNA/gold particles were bombarded onto these sites by a gene gun at 400 lb/in^2^ [35]. Animals were immunized with 10 shots (five per ear) of either wild type E7 expression construct or VIJns vector as control. This represents a theoretical 10 µg. Immunizations were done at 2–3 week intervals. A total of four immunizations were done rather than the normal three as we encountered problems coating the DNA/gold onto the tubing. One week after the final booster immunization, each rabbit was challenged with 10 µg wt H. CRPV DNA and each of the codon-optimized genomes, E7/8, E7/14, E7/18 and E7/22, at two sites per construct using the protocol discussed below. There were six animals per group.

### DNA challenge and monitoring of tumors

The CRPV plasmids were purified by cesium chloride ultracentrifugation and adjusted to 200 µg/ml in 1× TE buffer [36] for challenge on animals. For application of viral DNA, rabbits were sedated using Ketamine (40 mg/kg)/xylazine (5 mg/kg) anesthesia. Back skin of the animals was scarified with a scalpel blade to create an abrasion. Three days later, the wounded sites were lightly scratched with a scalpel blade to introduce nicks into the scabs. 10 micrograms of DNA in 50microliters of TE was applied to each site and the DNA was worked into the wound with a tuberculin syringe. [36]. Monitoring of papilloma outgrowth began two weeks later and continued until week 12.

### Statistics

Papilloma size was determined by calculating the cubic root of the product of length×width×height of individual papillomas in millimeters to obtain a geometric mean diameter (GMD). Data were represented as the means±SEMs (standard errors) of the GMDs for each test group. Statistical significance was determined by unpaired t-test comparison (P<0.05 was considered significant).
